# Effects of Climatic Conditions and Soil Properties on Cabernet Sauvignon Berry Growth and Anthocyanin Profiles

**DOI:** 10.3390/molecules190913683

**Published:** 2014-09-02

**Authors:** Guo Cheng, Yan-Nan He, Tai-Xin Yue, Jun Wang, Zhen-Wen Zhang

**Affiliations:** 1 College of Enology, Northwest A&F University, Yangling 712100, Shaanxi, China; E-Mails: berry713@163.com (G.C.); tina7088@yeah.net (Y.-N.H.); yue151@163.com (T.-X.Y.); 2 Center for Viticulture and Enology, College of Food Science & Nutritional Engineering, China Agricultural University, Beijing 100083, China; E-Mail: junwang1966@163.com; 3 Shaanxi Engineering Research Center for Viti-Viniculture, Northwest A&F University, Yangling 712100, Shaanxi, China

**Keywords:** Cabernet Sauvignon, climatic conditons, soil properties, berry growth, anthocyanin profiles

## Abstract

Climatic conditions and soil type have significant influence on grape ripening and wine quality. The reported study was conducted in two “Cabernet Sauvignon (*Vitis vinifera* L.V)” vineyards located in Xinjiang, a semiarid wine-producing region of China during two vintages (2011 and 2012). The results indicate that soil and climate affected berry growth and anthocyanin profiles. These two localities were within a distance of 5 km from each other and had soils of different physical and chemical composition. For each vineyard, the differences of anthocyanin concentrations, and parameters concerning berry growth and composition between the two years could be explained by different climatic conditions. Soil effect was studied by investigation of differences in berry composition and anthocyanin profiles between the two vineyards in the same year, which could be explained mainly by the different soil properties, vine water and nitrogen status. Specifically, the soils with less water and organic matter produced looser clusters, heavier berry skins and higher TSS, which contributed to the excellent performance of grapes. Compared with 2011, the increases in anthocyanin concentrations for each vineyard in 2012 could be attributed to smaller number of extreme temperature (>35 °C) days and rainfall, lower vine water status and N level. The explanation for higher anthocyanin concentrations in grape skins from the soils with less water and organic matter could be the vine status differences, lighter berry weight and heavier skin weight at harvest. In particular, grapes from the soils with less water and organic matter had higher levels of 3′5′-substituded, *O*-methylated and acylated anthocyanins, which represented a positive characteristic conferring more stable pigmentation to the corresponding wine in the future. The present work clarifies the effects of climate and soil on berry growth and anthocyanin profiles, thus providing guidance for production of high-quality wine grapes in different regions.

## 1. Introduction

Anthocyanins are an important group of flavonoids and the predominant pigments in red and black grape berries. After veraison, they accumulate in the grape skins via the phenylpropanoid biosynthetic pathway [1]. The anthocyanins in grapes are responsible for the colour of the corresponding wines, which is an important parameter used to evaluate wine quality [2]. Accumulation of anthocyanins in grape skins is influenced by many environmental factors such as light and temperature and nutrient supply [3]. In *Vitis vinifera* L. varieties, they occur mainly as glycosides and acylglucosides of five anthocyanidins: malvidin, petunidin, peonidin, delphinidin and cyanidin [4]. Monoglucosides exist as 3-*O*-glucosides in *V. vinifera* L. varieties, and the acylated anthocyanins are formed by the combination with coumaric or caffeic acid [5]. 

The concept of terroir, including the grape cultivar, always interacts with climatic conditions, soils, cultural practices and training systems, all of which influence the grape and wine quality [6]. Generally, cultivar, soil and climate are considered to be the three main components of terroir [7]. It is known that patterns of anthocyanins are controlled by strict genotype, and the anthocyanin profiles for each variety are relatively stable and their distribution varies considerably among different grape cultivars [8]. More recently, the wine industry has turned its attention to the factors of soil and climate. They point out the soil type may play an important role in determining wine characteristics and quality, resulting in different wine styles, even under the same mesoclimate [7]. In addition, other studies show that the influence of vintage on grape metabolic profiles is greater than the soil, where climatic characteristics such as the temperature and water balance are the main factors [9]. It should be noted that anthocyanin synthesis is depressed by excessively high temperatures with considering the specific circumstances [7]. Further research has revealed that the inhibition of temperature stress on berry secondary metabolites can be attributed to ABA-mediated actions [10]. Moreover, some research has determined that moderate temperatures encourage anthocyanin accumulation and alter partitioning, while others determined that high temperatures could be inhibitory to accumulation due to the differences in gene expression and chemical degradation of metabolites [7,11]. In addition to the effects of climate and soil, there is a great need to evaluate the influences of water deficits and berry exposure to sunlight on anthocyanin concentrations [12,13]. The water deficits enable anthocyanin concentrations to rise by both increasing content per berry and reducing fruit growth [14,15]. Generally, low level of light exposure could reduce flavonoids content, but increased light results in increased contents of anthocyanins and other flavonoids [16]. However, some authors have reported no change with different light treatments [17], or even that high light resulted in decreased anthocyanin concentrations [18].

Despite the fact that soil and climate are two important components of terroir, little experimental work concerning their influence on wine grape composition has been done in China. Therefore, in this work the effects of climate and soil on grape development and anthocyanin profiles were analysed. The soil effect was examined in two vineyards with distinct soil types, and climate effect was studied through the mesoclimate by inter-annual analysis. Specifically, the effects of soil components and climatic conditions were evaluated on: (1) vine water and nutrient status; (2) yield parameters and berry characteristics; (3) anthocyanin composition and concentrations. The present work was designed to study anthocyanin biosynthesis under different types of soils and climatic conditions. Special emphasis was put on the relationship between anthocyanin accumulation and environmental conditions. 

## 2. Results and Discussion

### 2.1. Climatic Conditions

**Figure 1:**
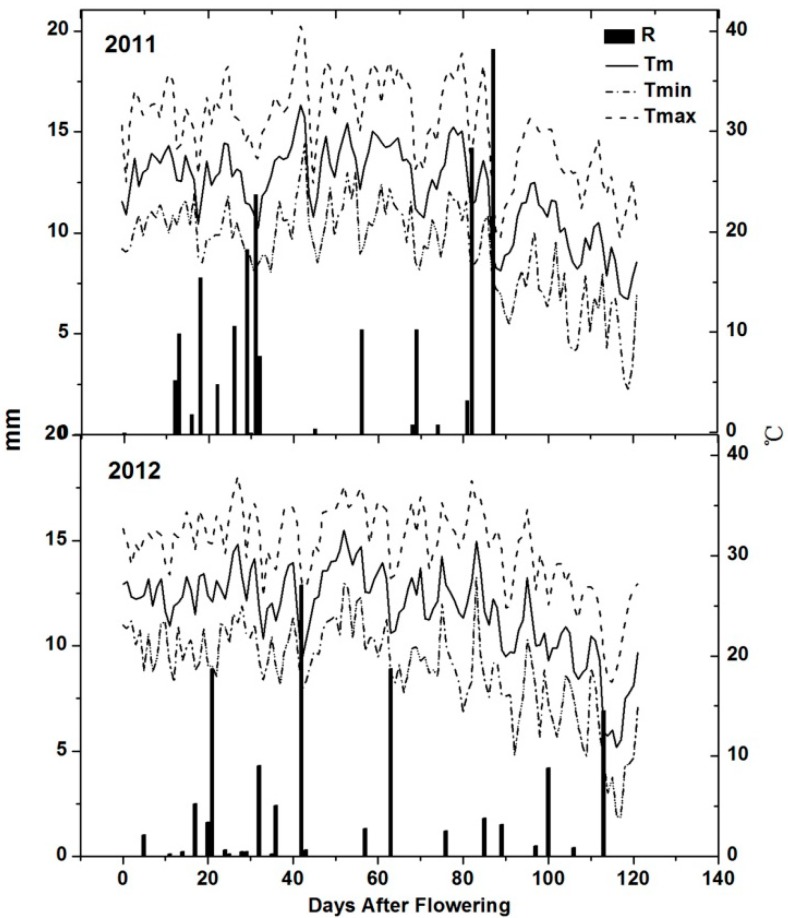
Evolution of mean, maximum and minimum daily temperature and rainfall from flowering to harvest in the study area (2011 and 2012). R, Rainfall; Tm, mean temperature; Tmax, maximum temperature; Tmin, minimum temperature.

The wine grape-growing regions of China display unique ecological conditions either from south to north or from east to west. The two vineyards in the present study were located in northwest China in a region with a semi-arid climate, with high biologically effective day temperatures, a big temperature difference between daytime and nighttime and low annual rainfall. All the meteorological data of 2011 and 2012 were obtained from the local meteorological administration. The two studied vineyards were within a radius of 5 km with a uniform mesoclimate. With respect to annual mean temperature and average maximum temperature, 2012 was warmer than 2011 (Table 1). With regard to the study period, the accumulated heat expressed as growing degree days (GDD, calculated from daily mean temperatures, base 10 °C) and sunlight duration in 2011 was lower than in 2012. Total rainfall, calculated from April to September, was 180.1 mm in 2011 and 103.6 mm in 2012 (Table 1). Regarding the seasonal evolution of temperature and rainfall (Figure 1), rainfall in 2011 was higher than in 2012 (mainly during 60–90 DAF), although vines did not get any rainfall at harvest (90–120 DAF) in 2011. According to the seasonal pattern during berry developmental phases (Figure 1), daily maximum temperature exceeded 30 °C for most of the summer period. A higher number of days with extreme temperatures (>35 °C) were observed in 2011 than in 2012 (23 and 13 days, respectively), mainly during expanding stage and veraison (0–90 DAF). Rainfall was on the rise from veraison (60–90 DAF) to harvest in 2012, but reduced gradually in 2011.

### 2.2. Soil Chemical and Physical Properties Analysis

The selected soils from two vineyards presented very different physical and chemical properties (Table 2), although both of the soils were classified as “slit loam” according to the texture classes in the soil survey manual. The soils from Yuanyi farm were richer in sand than those of Guangdong farm, especially in topsoil (0–20 cm). Specifically, the sand percentage was 41.99% at Yuanyi farm, and only 20.03% at Guangdong farm (Table 2). 

**Table 1:** Meteorological parameters for the growing season (April–October) and the ripening period (August–September) in the study area (2011 and 2012).

Year	Mean Temperature ( °C)			Average Maximum Temperature ( °C)			Average Minimum Temperature ( °C)			Growing Degree Days ( °C)			Sunlight Duration (h)			Rain (mm)		
	Aug-Sep	Apr-Oct	Year	Aug-Sep	Apr-Oct	Year	Aug-Sep	Apr-Oct	Year	Aug-Sep	Apr-Oct	Year	Aug-Sep	Apr-Oct	Year	Aug-Sep	Apr-Oct	Year
2011	22.1	20.3	8.2	28.7	26.8	13.6	16.1	14.6	3.4	740.9	2290.6	2290.6	627.4	2173.7	2899.0	41.2	180.1	217.8
2012	22.3	20.8	8.8	29.2	27.4	13.9	16.1	14.7	3.1	752.9	2356.2	2367.2	647.3	2235.8	3102.0	29.0	103.6	179.8

**Table 2:** Physical and chemical properties of the selected soils in two vineyards at different depths. Data are mean values of three replications.

Vineyards	Depth (cm)	Clay (%)	Slit (%)	Sand (%)	Textural Class	pH	EC (ms/cm)	CEC (cmol/kg)	Organic Matter (%)	Bulk Density (kg/m^3^)	Water Content (%)
Yuanyi farm	0–20	8.59b	49.40b	41.99a	Silt loam	7.9a	0.18b	16.40b	1.40b	1.49a	18.82b
	20–40	9.82b	61.66b	28.52a	Silt loam	7.8a	0.20b	13.18b	0.99b	1.65a	26.49b
	40–60	11.37a	54.13a	34.50a	Silt loam	8.1a	0.19b	12.82b	0.56b	1.41a	26.83b
	60–80	14.63a	49.87b	35.49a	Silt loam	8.0a	0.20b	12.46b	0.54b	1.50a	25.61b
	80–100	9.02a	55.72b	35.26a	Silt loam	7.9a	0.18b	11.74b	0.41b	1.55a	27.97b
Guangdong farm	0–20	15.99a	63.97a	20.03b	Silt loam	7.5b	0.39a	23.21a	6.38a	0.98b	29.55a
	20–40	11.45a	63.51a	25.04b	Silt loam	7.3b	0.54a	25.36a	8.42a	1.21b	44.04a
	40–60	8.30b	55.69a	36.01a	Silt loam	7.4b	0.56a	22.14a	6.85a	1.29b	38.89a
	60–80	9.17b	54.35a	36.48a	Silt loam	7.2b	0.98a	19.63a	5.02a	1.47b	36.13a
	80–100	9.68a	58.12a	32.20b	Silt loam	7.2b	0.66a	18.55a	3.69a	1.51b	33.74a

Values within a column followed by different letters differ significantly in the same depth between two vineyards (*t*-test, *p* < 0.05).

**Figure 2:**
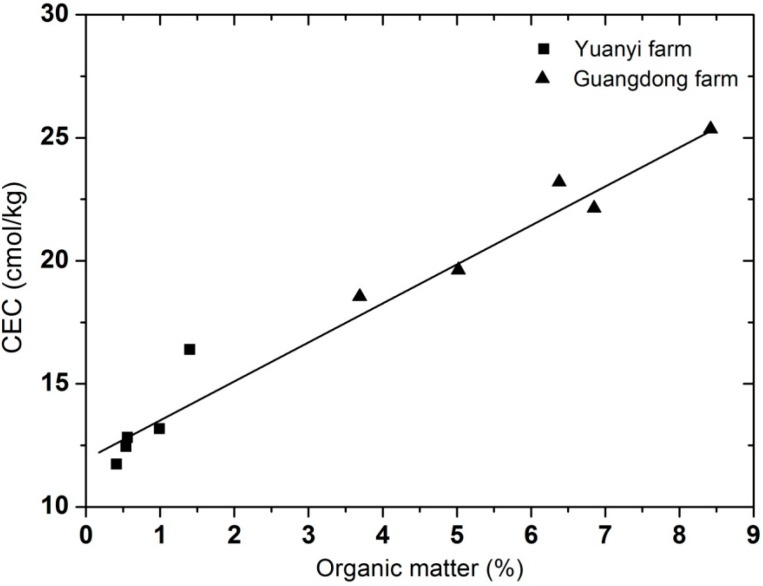
The relationship between cation-exchange capacity (CEC) and organic matter at Yuanyi farm and Guangdong farm. Data are mean values of three replications.

The soil pH at Yuanyi farm ranged from 7.8 to 8.1, which classified them as slightly alkaline soils, while the pH at Guangdong ranged from 7.2 to 7.5, which defined them as very slightly alkaline soils. At pH values above 5.0 to 5.5, grapevine performance should not be seriously impeded [19], and none of the soils in Yuanyi farm or Guangdong farm showed any signs of sodicity. Soil electrical conductivity (EC) is a parameter which represents the amount of salts in soils (soil salinity). In our study, the EC of soils from Guangdong farm was significantly higher than at Yuanyi farm at each soil depth (54%–80%). According to the classes of salinity and EC in the NRCS Soil Survey Handbook, all the selected soils from the two vineyards belonged to the non-saline (0<EC<2) class. The relationship between cation-exchange capacity (CEC) and organic matter at the two vineyards showed a positive correlation (Figure 2), and both of them decreased as the soil depth deepened (Table 2). As indicated in Table 2, the soils from Yuanyi farm had significantly higher bulk density at each depth than the Guangdong farm ones, since total pore space in sands is less than is silt or clay soils. For Guangdong farm, bulk density typically increased with soil depth since the subsurface layers contained less pore spaces and higher organic matter levels. Higher organic material may result in less compact soils. From the survey of bulk density, Yuanyi farm had more compact soils than Guangdong farm. Thus, the soils from Guangdong farm contained significantly higher percentages of organic matter than those of Yuanyi farm. In previous research, the organic matter content of soils in two vineyards located in Catalonia, Spain was 0.4%–2.0% [20]. Another report pointed out that soils with 0.7%–0.9% organic matter were considered to be poor, whereas those with 1.7%–1.8% organic matter were considered to be rich soils [21]. Thus, the organic matter content of soils in Guangdong farm was excessive (3.69%–8.42%) considering the general range of a rich soil. Nevertheless, the organic matter content of soils in Yuanyi farm was within a reasonable range (0.41%–1.40%). Water content of soils from Guangdong farm was significantly higher than at Yuanyi farm at each soil depth (17.2%–39.9%). Those could be attributed to organic matter increasing the water holding ability of soils directly and indirectly. On the other hand, higher bulk density consequently reduced the water holding capacity of soils. 

**Figure 3:**
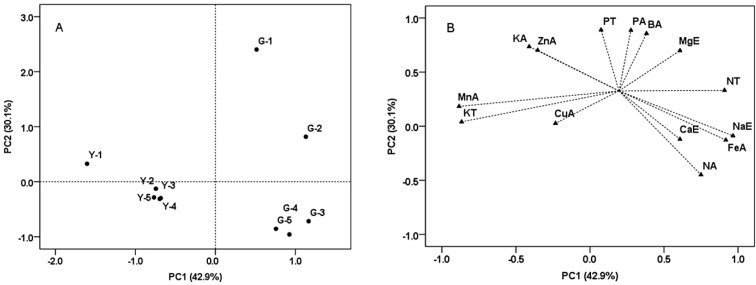
Discrimination of the soil samples from two vineyards by some kind of elements attributes and illustrated by the score (**A**) and loading (**B**) plots from principal component analysis (PCA).Y, Yuanyi farm; G, Guangdong farm; 1 = 0–20 cm; 2 = 20–40 cm; 3 = 40–60 cm; 4 = 60–80 cm; 5 = 80–100 cm; T, Total; E, Effective; A, Readily available.

Principal component analysis (PCA) of soil properties from the two vineyards was conducted by using the variables including total, effective and readily available elements variables (Figure 3). PC1 explained 42.9% of total variance, and was characterized by soils of all depths from Guangdong farm on the positive side. PC1 separated the soil samples of Yuanyi farm from Guangdong farm, and was mostly explained by total N, effective Na and available Fe having positive loadings. Specifically, the concentrations of these elements from soil samples were significantly higher in Guangdong farm (Table 3). Total K and available Mn had negative loadings, and their concentrations were significantly higher in soil samples from Yuanyi farm (Table 3). PC2 explained 30.1% of the variance and separated top soils (0–20 cm) of the two vineyards from the middle and bottom soils (40–100 cm). The top soils of two vineyards had abundant available elements such as P, Mn, Zn and B (Table 3).

**Table 3:** Chemical properties of the selected soils in two vineyards at different depths. Data are mean values of three replications.

Vineyards	Depth (cm)	Total Elements (%)			Effective Elements (mg/kg)			Available Elements (mg/kg)							
		N	P	K	Na	Ca	Mg	N	P	K	Cu	Fe	Mn	Zn	B
Yuanyi farm	0–20	0.04b	0.08a	1.83a	21.07b	3494.46b	140.62b	50.86b	10.10b	133.29b	0.75b	1.61b	1.01a	1.40a	0.47b
	20–40	0.07b	0.08a	1.85a	33.40b	3689.73b	219.12b	76.93a	3.09b	122.70a	0.85a	1.09b	0.59a	1.29a	0.11b
	40–60	0.04b	0.08a	1.80a	31.33b	3811.77a	233.70b	59.05b	1.45b	123.05a	0.75a	0.89b	0.65a	0.66a	Nd
	60–80	0.05b	0.07a	1.81a	39.48b	3760.86b	266.62a	53.24b	4.33b	152.14a	0.72b	0.83b	0.65a	0.38a	Nd
	80–100	0.07b	0.07a	1.85a	39.77b	3800.32a	295.58a	55.61b	4.36b	134.72a	0.76b	1.10b	0.91a	0.41a	Nd
Guangdong farm	0–20	0.30a	0.11a	1.73b	51.83a	3789.53a	525.98a	61.74a	75.42a	175.10a	0.85a	19.68a	0.58b	1.35b	2.51a
	20–40	0.40a	0.10a	1.34b	57.28a	3775.11a	490.15a	69.34b	18.25a	129.57a	0.58b	22.65a	0.45b	0.82b	1.03a
	40–60	0.30a	0.06a	1.40b	66.10a	3776.49b	334.47a	91.94a	5.68a	100.67b	0.61b	29.79a	0.41b	0.59b	0.21
	60–80	0.24a	0.06a	1.57b	63.41a	3824.79a	265.22a	86.13a	8.59a	78.92b	0.84a	33.92a	0.40b	0.35b	0.25
	80–100	0.19a	0.08a	1.66b	52.72a	3769.12b	219.11b	90.66a	8.93a	40.57b	0.85a	33.26a	0.39b	0.26b	0.75

Values within a column followed by different letters differ significantly in the same depth between two vineyards (*t-*test, *p* < 0.05). Nd, not detected.

### 2.3. Vine Water and Nitrogen Status

Ambient CO_2_ contains 98.9% of ^12^C isotope and 1.1% of ^13^C isotope. ^12^C is more easily used by the enzymes of photosynthesis in their production of hexoses. Therefore, the sugar produced by photosynthesis contains a higher rate of the ^12^C isotope than ambient CO_2_. This process is called ‘isotope discrimination’. When grapevines are submitted to water deficits, isotope discrimination is reduced due to stomatal closure [22]. Therefore, the ^12^C/^13^C ratio (so-called δ^13^C) allows a very sensitive detection of grapevine water status during grape ripening [23].

With respect to vine water deficit thresholds [24], there was no water deficit in vines from Guangdong farm during the two seasons (δ^13^C < −26). However, vines grown in Yuanyi farm showed weak water deficit for its higher δ^13^C values (−24.5 < δ^13^C < −26) corresponding to its soils with lower water-holding capacity compared with Guangdong farm (Table 4). Thus, the water content in soils affected the vine water status, which was confirmed by a significant negative correlation between δ^13^C and water content in soils (Table S1). Furthermore, N level was significantly lower in the vines of Yuanyi farm than at Guangdong farm. Soubeyrand *et al.* [3] used the chlorophyll content of the leaves to estimate the plant nitrogen status for the positive correlation between them. The chlorophyll content of the leaves from vines grown in Guangdong farm was significantly higher than that in Yuanyi farm. In addition, there was a positive correlation between N status of the plant*-*leaf Chlorophyll, N status of the plan*t-*organic matter in soils (Table S1). This confirmed that the higher nitrogen content in the soils of Guangdong farm led to an increase in nitrogen uptake and assimilation by the vines. Aside from other considerations, examination of these results showed the fact that there were differences in vine nitrogen and water status between the soils of the two vineyards. 

**Table 4:** Differences of vine water and nutrient status between Yuanyi and Guangdong farm during two seasons. Data are mean values of three replications at least.

Year	Vineyards	δ^13^C (‰)	N (%)	Leaf Chlorophyll
2011	Yuanyi farm	−25.34a	0.47b	45.01b
	Guangdong farm	−26.76b	0.54a	46.95a
2012	Yuanyi farm	−25.06a	0.46b	45.75b
	Guangdong farm	−26.51b	0.49a	46.54a

Values within a column followed by different letters differ significantly between two vineyards in the same year (*t-*test, *p* < 0.05).

### 2.4. Some Parameters of Vine, Shoot, Cluster and Berries from Two Vineyards in Two Vintages

Although there were no significant differences between the two vineyards regarding shoot number, yield, yield/pruning weigh and leaf area/yield in 2011 and 2012 (Table 5), vines grown in Guangdong farm had significantly longer shoots and less number of clusters per shoot than Yuanyi farm ones (Table 5). In addition, Yuanyi farm had significantly lighter and looser clusters compared with Guangdong farm (Table 5). From Table S1, there was a positive correlation between cluster compactness-organic matter in soils, cluster compactness-water content in soils. Thus, the clusters from the soils with less organic matter and lower water content would be characterized by their looser clusters, and this conclusion is verified in the discussion about anthocyanins. 

**Table 5:** Survey of some parameters about yields from two vineyards at harvest in 2011 and 2012. Data are mean values of three replications at least.

Years	Vineyards	Survey of Shoots			Survey of Clusters		Yield (ton/hectare)	Yield/Pruning Weigh (kg/kg)	Leaf Area/Yield (m^2^/kg)
		Average Shoot Length (cm)	Shoot Number/m	Cluster Number/Shoot	Cluster Weight (g)	Cluster Compactness (OIV rating)			
2011	Yuanyi farm	122b a	13.85a	1.80a	107.10b	4.00b	9.14a	5.49a	1.98a
	Guangdong farm	127a	14.29a	1.64b	111.22a	5.67a	8.45a	5.32a	2.08a
2012	Yuanyi farm	132b	13.55a	1.79a	107.97b	3.67b	10.48a	5.66a	2.03a
	Guangdong farm	136a	15.38a	1.58b	119.20a	5.33a	11.59a	5.78a	2.12a

Values within a column followed by different letters differ significantly between two vineyards in the same year (*t-*test, *p* < 0.05).

**Figure 4:**
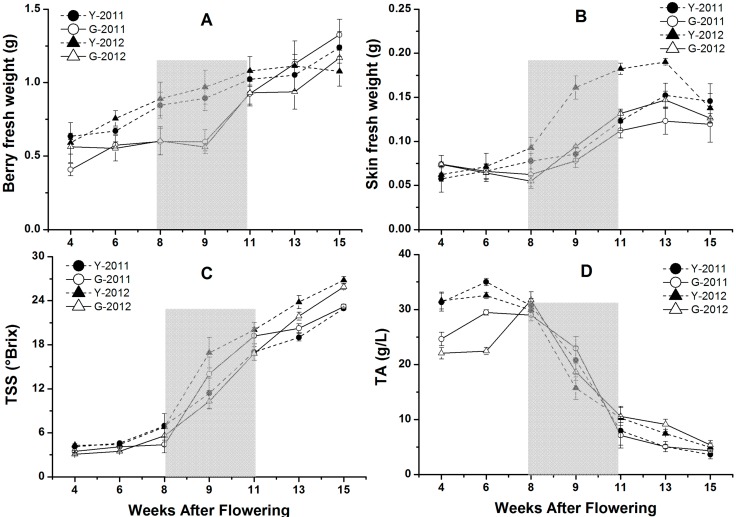
Some physical and chemical characteristics of berry changed with the grape development in two vineyards in 2011 and 2012. Data are mean values of three replications. Error bars show standard error (SE). Light grey background represents the phase of bunch turning colour (veraison) from 10% to 100% of coloured berries.

Some physical and chemical characteristics of berries were measured for grape samples from the two vineyards during different developmental stages (Figure 4). During the grape development in 2011 and 2012, fresh weight per berry increased until harvest in Yuanyi and Guangdong farm (Figure 4A). During the early stages of berry development, berries grew faster at Yuanyi farm than at Guangdong farm between 4 and 11 weeks after flowering (WAF). At harvest, fresh weight per berry in Guangdong farm was higher than Yuanyi farm in the two-year study. Overall, berries had a heavier weight in 2011 than in 2012. Skin weight per berry showed an increasing trend from 4 to 11 WAF, then decreased from 13 WAF to harvest (Figure 4B). 

Low nitrogen would be expected to cause a lower rate of shoot growth and chlorophyll formation [25]. The growth of the shoots is strongly affected by soil conditions and they grow longer where soils are more fertile and can hold more water [26]. In a previous report, cluster weight showed a negative correlation with the number of clusters per grapevine, because of the increasing berry numbers per cluster and weight caused by the decreasing cluster number [27]. Thus similar result patterns were seen in the two vineyards regarding shoot and cluster parameters as in previous research. Berry weight at Yuanyi farm was lighter in the soils with lower water content and weak water deficit for vines, which was consistent with a low water supply causing a reduction in berry weight [28]. In each vintage, the value of skin weight for Yuanyi farm was higher than for Guangdong farm. Previous research on deficit irrigation has shown an increase in skin weight [29], and a significant negative correlation between skin fresh weight and leaf Chlorophyll in our study (Table S1). Thus, the differences in the soil and vine parameters at Yuanyi and Guangdong farm make it easy to draw conclusions on the basis of these results. 

TSS exhibited a continuous increase from 4 to 15 WAF (Figure 4C). The maximum values of TSS for Yuanyi farm and Guangdong farm were 23.0 and 23.2 °Brix in 2011, but 26.8 and 25.9 °Brix in 2012. On the other hand, the changes of TA in juice of grapes from two vineyards in 2011 and 2012 are shown in Figure 4D. Grapes from the two vineyards in 2012 had higher TA than in 2011, and for each year, Guangdong farm had more TA in grape juice compared with Yuanyi farm. 

Adequate ripening may also be problematic in soils that are too wet [19]. While, in this study, TSS was equivalent in the two vineyards at harvest in 2011, the TSS of grapes from Yuanyi farm was higher than at Guangdong farm in 2012. A higher TA in the grapes from Guangdong farm was consistent with the other findings where canopies with excessive soil moisture had higher TA [30]. On the other hand, grapes with lower TSS and higher TA at the Guangdong farm were attributed to wetter soils, but this could also have been an effect of excessive organic matter.

### 2.5. The Concentrations of Anthocyanins in Grape Skins from Two Vineyards in 2011 and 2012

**Figure 5:**
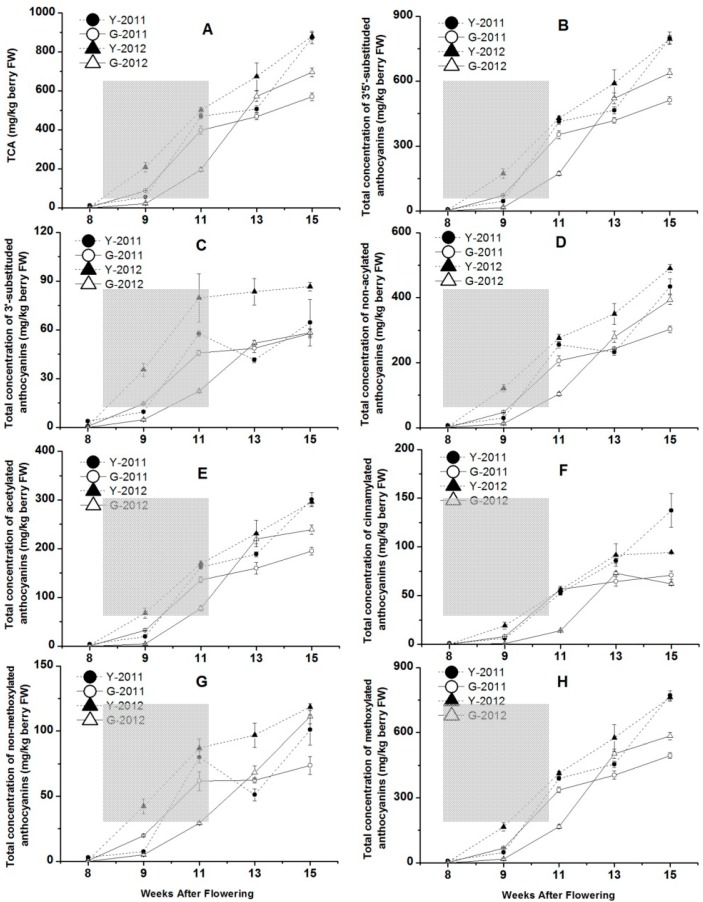
The concentration of total anthocyanins, 3′5′-substituded and 3′-substituded anthocyanins, non-acylated, acetylated and cinnamylated anthocyanins, non-methoxylated and methoxylated anthocyanins changed with the grape development in two vineyards in 2011 and 2012. Data are mean values of three replications. Error bars show standard error (SE). Light grey background represents the phase of bunch turning colour (veraison) from 10% to 100% of coloured berries.

The anthocyanins extracted from the grape skins during crushing, pressing, and fermentation are the major components responsible for red wine color [31]. The total concentration of anthocyanins (TCA) and each kinds of anthocyanin with different modifications showed continuous increases from 8 to 15 WAF (Figure 5A). The late stage of veraison (9 to 11 WAF) was a period of rapid accumulation for anthocyanin compounds. The maximum TCA values for Yuanyi farm were 873.3 mg/kg at harvest in 2011 and 880.4 mg/kg in 2012, but those for Guangdong farm were 568.9 mg/kg in 2011 and 695.2 mg/kg in 2012 (Figure 5A). In 2011 and 2012, grapes harvested from Yuanyi farm accumulated 35% and 21% more anthocyanins, respectively, than those from Guangdong farm, if anthocyanin content was expressed as concentration in fresh berry (mg/kg of berry fresh weight). This indicated that the effect of vintage was more significant in Guangdong farm when discussing TCA during the two seasons, while the regional differences always illustrated that grapes from Yuanyi farm could accumulate much more TCA. Meanwhile, TCA in grape skins is more variable than TSS (Figure 4C) between the two vineyards at each sampling point, although the evolution of anthocyanins was parallelled by the accumulation of sugars. 

In previous studies, some reported vintage variation [32], whereas others reported minimal influence of the season on anthocyanin accumulation [31]. Anthocyanin accumulation in grapes is light and temperature sensitive [32], so air temperatures might also have an influence on anthocyanin accumulation. Considering the climatic parameters such as mean temperature, the maximum and minimum temperature and growing degree days, 2011 was a cooler year with shorter sunlight duration and more rainfall compared with the same period in 2012 (Table 1), but extreme temperatures (>35 °C) were more frequent in 2011 than in 2012 (Figure 1). Although the lowest anthocyanin content was detected during the hottest year [20], this conclusion could not be verified in our study in each vineyard over two consecutive seasons. The total concentrations of anthocyanins at harvest in 2012 were higher than in 2011, especially in Guangdong farm, and their concentration increased by 22% (Figure 5).

Furthermore, the concentrations of anthocyanins expressed in mg/kg grapes were positively related to berry number and total skin surface per kilogram of grapes, resulting in the smallest berries being characterized by the highest content of anthocyanins [33]. In fact, exceedingly high temperatures (>35 °C) are particularly inhibitory to anthocyanin synthesis [32], so the increase of TCA in 2012 for each vineyard could be attributed to the decrease of berry fresh weight at harvest time (Figure 5), and less number of extreme temperature (>35 °C) days. Moreover, there was a significant positive correlation between TCA and δ^13^C, but negative correlation between TCA and N (Table S1). Thus, higher vine water status and N levels were also major causes for the low coloration of grapes in 2011. 

In this survey, the effect of the soils on vine behaviour was mediated through varying water content and nitrogen levels of the grapevines. With regard to the effect of water status, moderate and not severe water stress or drought stress have been reported to increase anthocyanin concentrations [34]. For the different growth rate of the skins and flesh responses to the change of water conditions, the mild water deficits increase the concentrations of skin tannins and anthocyanins through increasing skin weight, relative skin weight per berry, and therefore amounts of skin-localised solutes [35]. In the present study, Yuanyi farm had higher value of berry skins than Guangdong farm at harvest in each experimental season (Figure 4B). Furthermore, there was a significant positive correlation between TCA and skin fresh weight (Table S1). Research on terroir shows that a moderate nitrogen deficiency (like a mild water deficit) has been correlated with improved grape quality [36]. For the experimental samples, not only did Yuanyi farm have a faster speed of anthocyanin accumulation during berry development, but also a higher TCA at harvest in the two years (Figure 5). The above conclusions were also associated with lower water content of soils and vine water status at Yuanyi farm (Table S1).

F3′H (flavonoid 3′-hydroxylase) and F3′5′H (flavonoid 3′5′-hydroxylase) are involved in the biosynthetic pathway of cyanidin- and delphinidin-based anthocyanins, respectively. 3′5′-Substituted anthocyanins contain delphinidin, petunidin, malvidin anthocyanins and their derivatives. A higher accumulation of delphinidin-based anthocyanins in grape skins is expected to produce more intensely purple coloured berry and dark-colored red wine [37,38]. The concentrations of 3′5′-substituded anthocyanins showed an increasing trend during the developmental phase as well as TCA for each vineyard (Figure 5B). However, the concentration of 3′5′-substituded anthocyanins at Guangdong farm was 511 mg/kg, only 64% and 80% of that at Yuanyi farm at harvest in 2011 and 2012. On the other hand, the concentrations of 3′-substituded anthocyanins increased from veraison to harvest, although they remained at low ranges compared with 3′5′-substituded anthocyanins (Figure 5C). Additionally, 2012 showed higher concentrations of 3′5′- substituted anthocyanins than 2011. It was worth noting that the concentration of 3′-substituded anthocyanins in grape skins from Yuanyi farm in 2012 was the highest among all the developmental phases. From the analysis of correlation coefficients between variables, both of 3′5′-substituded and 3′-substituded anthocyanins showed significant positive correlations with skin fresh weight or δ^13^C, but significant negative correlations with vine N status (Table S1).

Considering further modification, two types of acylated anthocyanins are produced by acylation and cinnamylation in the grape skins of ‘Cabernet Sauvignon’. The concentrations of non-acylated, acetylated and cinnamylated anthocyanins accumulated and peaked at harvest stage for each vineyard (Figure 5D–F). For non-acylated and acetylated anthocyanins, the concentrations in the two vineyards in 2012 were higher than those in 2011 (Figure 5D–E), but the opposite was true for the concentrations of cinnamylated anthocyanins in each vineyard in 2011 and 2012 (Figure 5F). The concentration of each class of anthocyanins at Yuanyi farm was higher than at Guangdong farm at harvest, which could be associated with vine N status, cluster compactness and skin fresh weight (Table S1).

In addition, *O*-methylation of the 3′ position of the anthocyanidins cyanidin and delphinidin leads to the formation of peonidin and petunidin. Finally, *O*-methylation of positions 3′ and 5′ of delphinidin, leads to the formation of petunidin and malvidin. The methylated anthocyanins included peonidin, petunidin, malvidin anthocyanins and their derivatives. Because of their phenolic B ring substitution, peonidin and malvidin are relatively stable and represent the major anthocyanin pools in mature grapes [39]. The concentrations of methylated anthocyanins were 772 and 495 mg/kg in Yuanyi and Guangdong farm at harvest time in 2011, and 762 and 584 mg/kg in 2012, respectively (Figure 5G–H). Thus, the modification of anthocyanins influences their resulting wines by the colour, and grapes from the soils with less water content and organic matter are expected to produce more dark-colored red wine in the future (Table S1). 

### 2.6. Principal Component Analysis (PCA) of the Grape Samples from Two Vineyards at Harvest

**Figure 6:**
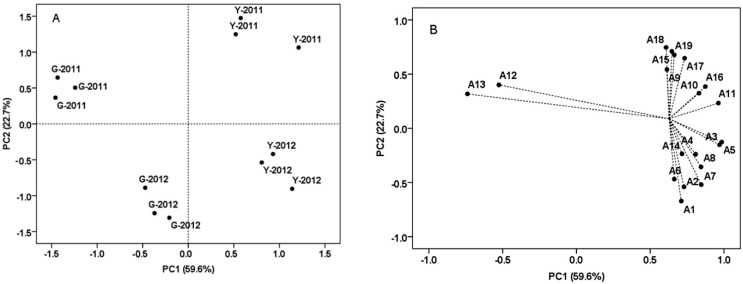
Discrimination of the grape samples from two vineyards by the concentration of individual anthocyanin detected at harvest in two vintages, and illustrated by the score (A) and loading (B) plots from principal component analysis (PCA). Each point means a biological replicate in plot A. Y, Yuanyi farm; G, Guangdong farm.

Previous research suggested that the anthocyanin fingerprint of grapes was related to cultivar and weather conditions of the growing season according to PCA [40]. In the present study, PCA was used to examine the effects of soil and vintage on the composition of anthocyanins (Figure 6). Nineteen different anthocyanins detected at harvest in two vintages were used as the variables, and each result contained three replicates, although malvidin-3-*O*-(6-*O*-caffeoyl)-glucoside was not detected in grape skins from Guangdong farm (Table 6). PC1 explained 59.6% of total variance, and characterized all grape samples from Yuanyi farm on the positive side. PC1 separated all the grape samples of Yuanyi farm from Guangdong farm, and could be mostly explained by malvidin-3-*O*-glucoside and malvidin-3-*O*-(6-*O*-acetyl)-glucoside having positive loadings. It is worth noting that malvidin-3-*O*-glucoside was the most prevalent anthocyanin and malvidin-3-*O*-(6-*O*-acetyl)-glucoside was the major acylated anthocyanin in all the grape samples from the two vineyards. Some similar observations are obtained from research conducted on ‘Merlot’ grape clusters in Prosser [41]. While petunidin-3-*O*-(6-*O*-caffeoyl)-glucoside and cyanidin-3-*O*-(6-*O*-coumaryl)-glucoside had negative loadings, and their concentrations were higher in grape samples from Guangdong farm (Table 6). PC2 explained 22.7% of the variance and separated grape samples of 2011 from 2012. In previous research, the percentages of dephinidin-3-*O*-glucoside in warm years was lower than in a relatively cool year [40]. In the present study, the grape samples of 2012 were abundant in dephinidin-3-*O*-glucoside, cyanidin-3-*O*-glucoside, dephinidin-3-*O*-(6-*O*-acetyl)-glucoside, cyanidin-3-*O*-(6-*O*-acetyl)-glucoside, and those of 2011 had higher concentrations of petunidin-3-*O*-(6-*O*-coumaryl)-glucoside, peonidin-3-*O*-(*trans*-6-*O*-coumaryl)-glucoside, malvidin-3-*O*-(*cis*-6-*O*-coumaryl)-glucoside and malvidin-3-*O*-(*trans*-6-*O*-coumaryl)-glucoside (Table 6). Most anthocyanins in grape skins from Yuanyi farm showed higher concentrations at harvest in each year, and for each vineyard, 2011 had higher concentrations of coumarylated anthocyanins than 2012, so by using the concentrations of individual anthocyanin detected at harvest we could clearly discriminate the effects of vineyard and vintage. 

**Table 6:** The concentration of individual anthocyanin (mean ± se; n = 3) detected in grape skins of “Cabernet Sauvignon” from two vineyards at harvest in 2011 and 2012.

Anthocyanins	2011		2012	
	Yuanyi farm (mg/kg berry FW)	Guangdong farm (mg/kg berry FW)	Yuanyi farm (mg/kg berry FW)	Guangdong farm (mg/kg berry FW)
dephinidin-3-*O*-glucoside (A1)	52.31 ± 3.7	37.65 ± 1.28	66.83 ± 0.86	63.20 ± 2.48
cyanidin-3-*O*-glucoside (A2)	6.97 ± 0.52	6.24 ± 0.27	8.49 ± 0.75	7.31 ± 0.21
petunidin-3-*O*-glucoside (A3)	42.36 ± 3.09	31.94 ± 1.61	43.40 ± 1.35	38.92 ± 1.29
peonidin-3-*O*-glucoside (A4)	28.71 ± 2.22	23.47 ± 1.39	39.28 ± 2.22	25.05 ± 1.44
malvidin-3-*O*-glucoside (A5)	302.77 ± 14.46	201.11 ± 6.17	332.49 ± 10.28	259.38 ± 10.72
dephinidin-3-*O*-(6-*O*-acetyl)-glucoside (A6)	24.94 ± 5.45	17.73 ± 5.19	28.22 ± 4.10	27.84 ± 3.43
cyanidin-3-*O*-(6-*O*-acetyl)-glucoside (A7)	5.45 ± 0.95	2.45 ± 0.18	7.91 ± 0.51	6.13 ± 0.65
petunidin-3-*O*-(6-*O*-acetyl)-glucoside (A8)	17.74 ± 1.76	12.51 ± 1.01	18.24 ± 0.49	17.95 ± 0.92
dephinidin-3-*O*-(6-*O*-coumaryl)-glucoside (A9)	7.67 ± 1.42	4.18 ± 0.40	4.98 ± 0.67	4.88 ± 0.47
peonidin-3-*O*-(6-*O*-acetyl)-glucoside (A10)	18.20 ± 0.86	13.99 ± 0.51	19.32 ± 0.34	12.58 ± 0.58
malvidin-3-*O*-(6-*O*-acetyl)-glucoside (A11)	234.36 ± 8.87	148.40 ± 2.63	221.82 ± 3.50	174.58 ± 5.01
petunidin-3-*O*-(6-*O*-caffeoyl)-glucoside (A12)	0.49 ± 0.15	1.07 ± 0.28	0.55 ± 0.00	0.31 ± 0.01
cyanidin-3-*O*-(6-*O*-coumaryl)-glucoside (A13)	2.05 ± 0.21	4.39 ± 0.54	2.00 ± 0.74	2.00 ± 0.76
malvidin-3-*O*-(6-*O*-caffeoyl)-glucoside (A14)	0.38 ± 0.10	Nd	1.21 ± 0.05	Nd
petunidin-3-*O*-(6-*O*-coumaryl)-glucoside (A15)	6.28 ± 0.43	3.75 ± 0.23	4.36 ± 0.07	3.60 ± 0.17
peonidin-3-*O*-(*cis*-6-*O*-coumaryl)-glucoside (A16)	0.65 ± 0.05	0.41 ± 0.01	0.64 ± 0.03	0.38 ± 0.01
peonidin-3-*O*-(*trans*-6-*O*-coumaryl)-glucoside (A17)	12.88 ± 1.99	6.85 ± 0.39	8.95 ± 0.32	4.85 ± 0.19
malvidin-3-*O*-(*cis*-6-*O*-coumaryl)-glucoside (A18)	4.88 ± 0.41	2.62 ± 0.16	3.65 ± 0.22	2.31 ± 0.09
malvidin-3-*O*-(*trans*-6-*O*-coumaryl)-glucoside (A19)	102.01 ± 16.81	47.64 ± 2.77	68.04 ± 0.60	43.95 ± 1.22

Each value represents the mean of three replicates and their standard. Nd, not detected.

## 3. Experimental Section

### 3.1. The Experimental Site and Plant Material

The field experiments were performed at two commercial “Cabernet Sauvignon” vineyards (Yuanyi farm and Guangdong farm) over two seasons (2011 and 2012). They are both located in Manas County (belonging to Shihezi City), the wine-producing region of Xinjiang, China. Yuanyi farm is located at 44°17′55′′ North, 86°12′2′′ East and at a altitude of 475 m, and Guangdong farm is located at 44°18'58′′ North, 86°13′1′′ East and at a altitude of 470 m. The growth area of ‘Cabernet Sauvignon’ was 2000 hectares, which accounted for around 80% of the wine grape cultivation in this county. The vines were planted in 2000, furrow irrigated, and grown on their own roots in a north–south row orientation. All the vines were trained to a slope trunk with a vertical shoot positioning trellis system, which we called Modified VSP (M-VSP) spaced at 2.5 m × 1.0 m (Figure S1), with a spur-pruned cordon retaining 15 nodes per linear metre. Crop load was normalized to approximately 30 bunches per plant. Grapevines in each vineyard were replicated on 45 vines, arranged in three consecutive rows.

The vines were managed according to industry standards for nutrition and pest management in the two vineyards. As in the preceding years, no nitrogen fertiliser was added in 2011 and 2012, hence vine nitrogen uptake was mainly from mineralization of organic matter. Pests and diseases were managed with application of lime sulphur, potassium dihydrogen phosphate and fertilizer containing iron and zinc in May and June, but diniconazole, mancozeb, and imidacloprid were used in July and August. In either vineyard, the management was the same, and there were no artificially induced differences between the two vineyards. 

### 3.2. Meteorological Survey

All the meteorological datas of 2011 and 2012 were obtained from the local meteorological administration. Sunlight duration (h), temperature (°C) and rainfall (mm) were recorded daily. Both of the studied plots had a similar mesoclimate, since these two localities were within a distance of 5 km from each other and they had very similar topographic characteristics (altitude, slope and orientation). 

### 3.3. Soil Sampling and Analysis

The chosen soils belonging to the two vineyards were collected from five different depths: 0–20 cm, 20–40 cm, 40–60 cm, 60–80 cm, 80–100 cm. The 10 soil sampling points were in a Z-shape, and 500 g of soil was collected from each depth and point, maintaining a distance of 100 cm away from the trunk. Then, all the soils from the same depth were mixed and 1 kg was retained for every sample based on “quartering”. A Mastersizer 2000E laser particle size analyzer was used in the determination of physical properties of the soil samples from the two vineyards. The soil texture sorting method followed the criteria of the Soil Survey Manual [42]. The soil bulk density and water content were measured and calculated by the cutting ring method, air-dried and then heated at 105 °C till they reached constant weight. The bulk density was obtained from the ratio between the dried mass and the volume of the cylinder, and more compacted soils with less pore space will have much higher bulk density .While water content of soils was calculated by the difference between the fresh and dried mass. Organic matter, cation exchange capacity (CEC) and pH of soil samples were measured by the potassium dichromate volumetry, EDTA-ammonium salt and potentiometry methods, respectively. On the other hand, nutrient elements in soils were determinated by plasma spectrometry (Vista-AX, Varian, Palo Alto, CA, USA) after extraction and digestion. 

### 3.4. Vine Water and Nutrient Status

For each vineyard, δ^13^C measurements were carried out on three individual samples of grape at harvest using an Isotope Ratio Mass Spectrometer (Thermo Fisher Scientific Inc., Waltham, MA, USA). The ^12^C/^13^C ratio in the sample is compared to that in an international standard, the so-called PDB standard which is a rock in which this ratio is particularly stable. The results vary from −20‰ (severe water deficit stress) to −27‰. δ^13^C are well-correlated to stem water potential [24]. 

Berry total nitrogen content can be used as a physiological indicator of vine nitrogen status [36]. Vine nutrient status was assessed on berries collected from the target vines at harvest time in the 2011 and 2012 vintages. The total nutrient contents were analysed using an elemental analyzer (Flash EA1112 HT, Thermo Fisher Scientific Inc., Waltham, MA, USA). Previous research has revealed that the chlorophyll content of the leaves is closely related to the N status of the plant [3]. Thus, the leaf chlorophyll content was measured non-destructively on leaves using a Minolta-502 dual wavelength chlorophyll (SPAD) meter (Minolta Co. Ltd, Osaka, Japan). In addition, thirty random measurements were made across each farm to give an average value used to indicate the vine N status.

### 3.5. Yield and Grape Composition

The berries were collected from 4 weeks after flowering till commercial harvest, and the sampling dates were scheduled at 2 week intervals. Forty five vines were selected on the basis of uniformity of shoot growth and cluster development in each vineyard, and the same plants were used at each sampling point. At veraison, the sampling points were increased at the stages of 10%, 50% and 100% coloured berries in both seasons. The sampling schedule in 2012 spanned the same developmental stages as in 2011. Harvest dates were determined by the cooperating winery. All the grape samples were harvested at technological ripeness, 23th and 24th Sep for Yuanyi farm and Guangdong farm in 2011, and 20th and 24th Sep for Yuanyi farm and Guangdong farm in 2012. For all the shoots from 10 vines, leaves were counted and the main and lateral leaves in each vineyard were measured by the portable leaf area meter (Yaxin-1242, Beijing, China) at veraison in the two seasons. On the commercial harvest day, average shoot length, shoot number per meter, cluster number per shoot were recorded for description of growth potential. Each survey was carried on 10 vines as parallel experiments. In each year, at the end of October, canes from 45 representative vines per vineyard were pruned and weighed to estimate the annual vine growth. These data was then used to calculate the yield-to-pruning weight ratio (kg/kg). Vine balance was also assessed by calculating the total leaf area-to-yield ratio in both vineyards.

Yield data were collected by hand-harvesting fruit from vines of each replicate on the commercial harvest day (18th Sep, 2011 and 20th Sep, 2012) in the two years. Bunches per vine and yield per vine were recorded, from which average bunch weights were determined. After clusters were weighed and compactness described according to OIV rating [43], 300 berries (100 per replicate) were then randomly separated from the pedicle of each vineyard using scissors, ensuring that berries were taken from all parts of the clusters. Berry samples (150, 50 per replicate) were used for determination of berry weight, then another 150 berry samples (50 per replicate) were crushed in a hand press through two layers of cheesecloth. Berry skins were obtained by carefully removing seeds and mesocarp. Berry skins were rinsed in deionised water, then were weighed after blotting excess water. Total soluble solids (TSS) was measured using a PAL-1 Digital Hand-held "Pocket" Refractometer (Atago, Tokyo, Japan). Titratable acidity (TA) was measured by titration with NaOH to the end point of pH 8.2 and expressed as tartaric acid equivalent. Each experiment was carried out in triplicate for berry and cluster samples from the two vineyards.

### 3.6. Extraction of Anthocyanins in Grape Skins

Anthocyanin analyses were done on frozen grapes after removing the stems. Three replicates of samples of grapes (80 berry per replicate) were selected for each developmental stage. While the grapes were still frozen, skins were separated from the pulp. Berry skins were frozen, crushed and then freeze-dried at −40 °C. Both the wet and dry weights were recorded.

The extraction of anthocyanins in grape skins was carried out according to the previously published method of He *et al.* [44]. Grape skin powder (0.50 g) was immersed in methanol (10 mL) containing 2% formic acid. This extraction was performed with the aid of ultrasound for 10 min, and then the mixture was shaken in the dark at 25 °C for 30 min at a rate of 150 rpm. The homogenate was centrifuged at 8,000× *g* for 10 min and the supernatant was collected. The residues were re-extracted four times. All the supernatants were mixed, concentrated to dryness using a rotary evaporator and then redissolved in 10 mL of solvent mixed with 90% mobile phase A and 10% mobile phase B (see below). The resulting suspensions were filtered through 0.22 μm filters (Micro Pes, Membrana, Wuppertal, Germany) prior to HPLC-MS analysis. Each sample was subjected to three independent extractions from three biological repeats.

### 3.7. Chemicals and Standards

The standard, malvidin-3-*O*-glucoside was purchased from Sigma-Aldrich Co. (St. Louis, MO, USA). HPLC grade methanol, formic acid and acetonitrile were purchased from Fisher (Fairlawn, NJ, USA). Analytical grade methanol and formic acid were purchased from the Beijing Chemical Reagent Plant (Beijing, China). 

### 3.8. HPLC-MS Analyses of Anthocyanins

An Agilent 1100 series LC-MSD trap VL (Agilent, Santa Clara, CA, USA), equipped with a G1379A degasser, a G1312BA QuatPump, a G1313A ALS, a G1316A column, a G1315A DAD and a Kromasil 100–5 C18 column (250 × 4.6 mm, 5 μm) was used for anthocyanins detection. A flow rate of l mL/min at ambient temperature was used. Solvent A was 2% (v:v) formic and 6% acetonitrile in water, and solvent B was acetonitrile containing 2% formic acid and 44% water. Proportions of solvent B varied as follows: 1–18 min, 10% to 25%; 18–20 min, 25%; 20–30 min, 25% to 40%; 30–35 min, 40% to 70% and 35–40 min, 70% to 100%. Injection volumes were 30 μL, and the detection wavelength was 525 nm. The column temperature was 50 °C. MS conditions were as follows: Electrospray ionisation (ESI) interface, positive ion model, 30 psi nebulizer pressure, 12 mL/min dry gas flow rate, 300 °C dry gas temperature, and scans at *m/z* 100–1500. Anthocyanins were quantified at 525 nm as malvidin-3-*O*-glucoside using calibration curves obtained within a concentration range between 0.5 and 500 mg/L, with linear correlation coefficients greater than 0.999 in the two years. Both standards and samples were determined in triplicate.

### 3.9. Statistical Analysis

Significant differences were determined at *p* < 0.05, according to independent *t-*test. Principal component analysis (PCA) of soil elements and anthocyanins in grape skins was used to achieve a obvious discrimination of different soil depths, regions and vintages. Statistical analysis was performed by SPSS (SPSS Inc., Chicago, IL, USA) for Windows, version 20.0. All the figures were drawn using the Origin 8.0 software. 

## 4. Conclusions

In the present research,, the effects of soil and climatic conditions were studied on ‘Cabernet Sauvignon’ grapes in two vintages (2011 and 2012). The results showed that the soils with less water and organic matter produced looser clusters, heavier berry skins and higher TSS, which contributed to the excellent performance of the grapes. Compared with 2011, the increased anthocyanin concentrations in 2012 for each vineyard could be attributed to a lesser number of extreme temperature (>35 °C) days and rainfall, lower vine water status and N level. The higher anthocyanin concentrations in grape skins from the soils with less water and organic matter were explained by vine status differences, lighter berry weight and heavier skin weight at harvest. In particular, grapes from the soils with less water and organic matter had higher levels of 3′5′-substituted-*O*-methylated- and acylated anthocyanins, which represented a positive characteristic responsible for more stable pigments in the corresponding wine in the future. In summary, the soils with less water and organic matter produce "Cabernet Sauvignon" grapes with much better quality in some important aspects of winemaking quality, such as berry characteristics and anthocyanin profiles.

## Supplementary Materials

Supplementary materials can be accessed at: http://www.mdpi.com/1420-3049/19/9/13683/s1.

## Supplementary Files

Supplementary File 1
