# Supplementary material for: Effects of Climatic Conditions and Soil Properties on Cabernet Sauvignon Berry Growth and Anthocyanin Profiles
Source: Molecules. 2014 Sep 2;19(9):13683–703. doi: 10.3390/molecules190913683 (PMC6271934; doi:10.3390/molecules190913683)
Supplement: Supplementary File 1 [file molecules-19-13683-s001.pdf]

Supplementary File

Figure S1. Sketch of Slope trunk with vertical shoot positioning training system (M-VSP).

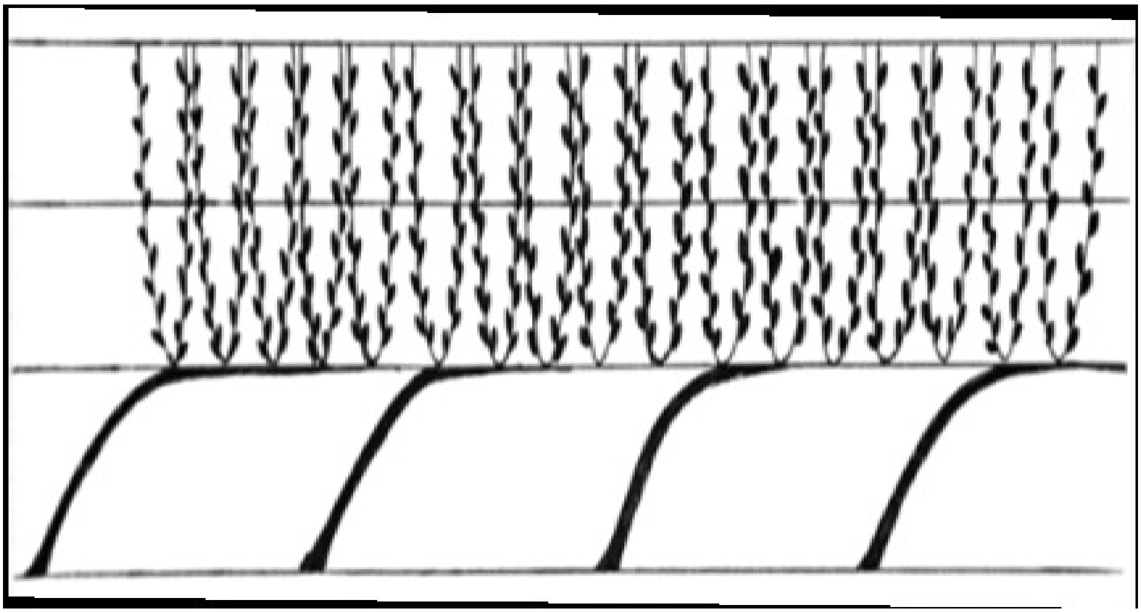

Table S1. Correlation coefficients between some variables on ‘Cabernet sauvignon’ grape from two vineyards in 2011 and 2012. A, Total concentrations of anthocyanins; B, 3'5'-substituded anthocyanins; C, 3'-substituded anthocyanins; D, non-acylated anthocyanins; E, acetylated anthocyanins; F, cinnamylated anthocyanins; G, non-methoxylated; H, methoxylated anthocyanins; I, berry fresh weight; J, skin fresh weight; K, cluster weight; L, cluster compactness; M, $\delta^{13}C$; N, N status of the plant; O, Leaf chlorophyⅡ; P, organic matter in soils; Q, water content in soils.

Variant	B	C	D	E	F	G	H	I	J	K	L	M	N	O	P	Q
A	0.999**	0.718	0.950*	0.998**	0.749	0.749	0.996	−0.677	0.951*	−0.551	−0.972*	0.968*	−0.961*	−0.92	−0.939	−0.939
B		0.69	0.946	0.999**	0.753	0.755	0.994**	−0.67	0.956*	−0.527	−0.961*	0.956*	−0.965*	−0.927	−0.928	−0.928
C			0.821	0.673	0.281	0.63	0.703	−0.788	0.515	−0.547	−0.825	0.825	−0.69	−0.452	−0.746	−0.746
D				0.934	0.506	0.894	0.92	−0.872	0.809	−0.375	−0.924	0.916	−0.979*	−0.754	−0.834	−0.834
E					0.774	0.736	0.995**	−0.645	0.965*	−0.541	−0.959*	0.955	−0.957*	0.939	−0.933	−0.933
F						0.155	0.803	−0.02	0.908	−0.776	−0.743	0.749	−0.567	−0.938	−0.846	−0.846
G							0.683	−0.951*	0.555	0.081	−0.655	0.640	−0.900	−0.487	−0.505	−0.505
H								−0.611	0.969*	−0.62	−0.978*	0.976*	−0.93	−0.945	−0.964*	−0.964*
I									−0.425	−0.011	0.648	−0.636	0.816	0.344	0.458	0.485
G										−0.627	−0.909	0.908	−0.86	−0.996**	−0.932	−0.932
K											0.696	−0.71	0.306	0.644	0.804	0.804
L												−1.00**	0.892	0.877	0.98*	0.98*
M													−0.883	−0.877	−0.983*	−0.983*
N														0.814	0.811	0.811
O															0.916	0.916
P																1.00**

** significant at 0.01 level, * significant at 0.05 level.
